# Editoral and Miscellaneous

**Published:** 1862-06

**Authors:** 


### EDITORIAL.

*The Sanitary Commission.*—An eastern contemporary seems to think, as we used to before we had "farther light," that the Government officials of the medical department have done their duty in furnishing medicine and hospital supplies. It may be so at the East, where millions upon millions have been lavished upon political favorites in contracts of every conceivable sort, including straw hats, linen pantaloons, herring, etc., etc.; but "out West" it has been different. The Government officials entrustsd with this matter have been derelict to the last extreme. Hundreds of lives would have been lost, and thousands would have experienced all the horrors of inattention, neglect, and we might almost say of starvation, if it had not been for the well directed efforts of the western agents of this benevolent association.

Whilst it would seem almost invidious to particularize, we cannot forbear especially mentioning the name of the Hon. Mark Skinner, Chairman of the Chicago Sanitary Commission, a distinguished citizen, and, we are proud to say, a leading Trustee of Rush Med. College, as peculiarly worthy of honor for the efforts which he has put forth and the success which has attended them.

Occasionally there is no doubt these supplies have been misappropriated or even wasted, of which certain startling evidences have been reported, but nevertheless the bulk, forming an enormous aggregate, has reached its destination and filled a *hiatus*, previously *valde deflendus.*

---

*Surgeon J. B. Porter.*—The newspaper readers must have noticed certain grave charges against Surgeon Porter, U. S. A., in charge of the General Hospital at Alexandria. Correspondents of the N. Y. *Tribune*, and other sensation newspapers, alleged brutality of treatment, and neglect of his patients. A Court of Inquiry was promptly convened, and the matter thoroughly investigated. Surgeon Porter, with conscious pride of integrity, scorned to introduce a single witness. His accusers were convicted out of their own mouths, and had the mortification of having Surgeon Porter, not only triumphantly acquitted, but expressly complimented in strong terms by the Court.

The only charge partially sustained had reference to a temporary bad quality of the bread supplied—a thing proven to have been no fault of his. We compliment this honorable, high-minded, and distinguished officer on his complete vindication.

---

*Foreign Correspondence.*—Dr. Edward L. Holmes, of this city, now visiting Europe for the further cultivation of the specialties to which he has largely devoted his attention, promises us a series of letters during his absence, which will appear from time to time in the JOURNAL.

Dr. Holmes having some years since spent considerable time in the prosecution of his favorite branches of professional study in the Continental hospitals, having already a large personal acquaintance, and being familiar with the German and French languages, will have peculiar facilities in his present visit, which we hope to share with our readers in results.

In his absence we venture to write, what we should not dare to write were he here, that upon excellent native abilities, cultivated by liberal education, he has engrafted large general professional attainment, with an exceedingly minute and comprehensive familiarity with diseases of the Eye and Ear.

Earnestly opposed as we are to specialism in practice, we cannot object to it where, as in the case of Dr. Holmes, it is not exclusive but based upon general professional acquirement.

*Camp Diarrhœa.*—One of our professional friends recently visited the army of the Union in a volunteer capacity. He found an Illinois regiment deprived of one of its surgeons by death and of the other by disease. It had no medicines or hospital supplies of any sort whatever. It had *three hundred and forty-nine* on its sick list. The prevalent disorder was diarrhœa. Undaunted by the difficulties before him, he went into the forest, gathered white oak bark, the root of the wild black berry, and managing to get some Capsicum and Carbonate of Ammonia, almost surreptitiously, he made a decoction of the first three and added the last. With "only this and nothing more" the sick list was reduced in a single week to *eighty.*

General Grant, who was suffering from the same difficulty, was relieved at once by the same remedy. The hint may be worth something to the medical attendants of the grand army. We call upon the Dr. to "relate his experience," over his own signature. The facts have been given us from a most reliable authority.

---

*A System of Surgery; Pathological, Diagnostic, Therapeutic, and Operative, by Samuel D. Gross, M. D., Professor of Surgery in the Jefferson Medical College of Philadelphia, etc. Illustrated by twelve hundred and twenty-seven Engravings. Second Edition. Much enlarged and carefully revised. In two volumes. Blanchard & Lea.* 1862.

We are gratified to be able to announce a new edition of this Cyclopædia of Surgery. Considering the large size of the work and its expensiveness, the extremely rapid sale and its exhaustion of an entire edition, not only proves the value of the work, but speaks well for the intelligence of American surgeons. The Second Edition bears evidence of careful revision, on nearly every page; and the whole sections of new matter prove with what care and pains-taking the distinguished author has labored to render his work worthy of the patronage of the profession. We cannot enter into even a specification of the additions which have been made, but will simply add that the work is still the most complete exponent of the science and art of surgery in our language.

We borrow from a contemporary the above brief notice of what we are certain must be a work meriting fully the encomium contained.

The Editors were notified of the forwarding of the work to this JOURNAL. Even the errand boy, who thinks he delivered it, has appeared on the stage, but the volumes we can lament for, as Rachel for her children, because "they are not."

The books were delivered, but not to the Editors or their representatives. "There is a bird i' the air" which whispers, (if it be a bird, and a bird ever whispers,) *who* is enriched by their surreptitious possession. He needs them, yet remember, Friend, *multitudo librorum sæpe est nubes testium ignorantiæ possessoris*—and you need no witnesses of that transparent fact in your case. You may eat the volumes, an' yon wish, but we fervently trust that they may prove as bitter in the belly of your conscience (if you have a conscience), as did the little book masticated in the Apocalypse. If you have a Bible, Friend, look up the allusion—a little Bible at all events will be (and long before this would have been) excellent in your case. Finally, may ascarides tease you, and calculus "gravel" you, and dyspepsia plague you, and scabies stick to you, until you send back those books. We will reward you for this virtue *in extremis*, with a bottle of cocoaine warranted pure, and "no questions asked."

---

*The Principles and Practice of Obstetrics. By Gunning S. Bedford, A. M., M. D., Professor, &c., &c. Second Edition. Carefully revised. New York: William Wood, 389 Broadway.* 1862. *8vo., pp.* 763.

This book has had a marvelous success, and yet we need scarcely say, not beyond its deserts. Not only is the first edition already exhausted, but it has enjoyed the rare distinction of having been translated and published in several foreign countries, where it has been received with high and almost universal commendation.

It takes rank at once as a standard work and authority in the department which it represents.

It is thoroughly readable as well as scientific—the author recognizes the fact, of which too many are oblivious, that *stilts* are not necessary to an impressive style. It is systematic, without being cramped or rigid. It is comprehensive, without being tedious. It is learned and exact, without being pedantic and finical. In his general views the author is judicious, sound and, what we especially delight to see, conservative. He knows how to acknowledge the general truth that a "meddlesome midwifery is bad," and yet with perfect consistency to direct at suitable times prompt and even heroic interference. The sentences on the abuse of embryotomy we commend especially to the earnest consideration of all junior (and a good many senior) practitioners. This latter feature of his work we rejoice to see especially commended by foreign reviewers.

Prof. Bedford evinces, it is not out of place to observe, throughout his volume, an appreciation of the true feminine character, and the relation of the accoucheur to his suffering patient, every way indicative of that delicacy and refinement which should distinguish the professional gentleman.

We predict that the vast labor, extensive research, enthusiasm and ability which Brof. Bedford has lavished upon this book, will carry it through, not only this, the second, but many subsequent editions.

---

*Chicory in Coffee.*—M. Lawey, referring to the great efficacy of good coffee as a substitute for brandy in soldier's rations, its less fugacious qualities as a stimulant, its probable prophylactic energy against malaria, its powers for counteracting the physiological effects of heat, and tendency to restrain alvine fluxes, calls attention to the fact that chicory, with which it is commonly largely adulterated, has none of these qualities, and that being laxative, it favors the occurrence of diarrhœa and dysentery. The point seems worthy of investigation, so that when soldiers by a somewhat questionable philanthropic or finical policy, are supplied with coffee instead of spirit rations, they may not be cheated of this even, and then poisoned by chicory or other knavish substitution.

---

*Secale Cornutum in Delirium Tremens.*—Several cases of recovery after the use of Ergot in Delirium Tremens have been recently reported. A writer in the *Am. Med. Monthly*, who says, however, that he has employed it in but one case, admits having been impressed by the apparent power of the remedy. Ice-water baths, Digitalis, Morphia in large doses, Alcohol ditto, and indeed a half score of other agents have each their advocates among late writers. But Delirium Tremens is cured by no drug. It is only met by restoring depraved or lost nutrition.

---

*Fucus Vesiculosus as a Remedy for Obesity.*—M. Duchesne Duparc strongly recommends this plant as a means of diminishing an uncomfortable tendency to obesity. He reports three cases in which its use was attended by a decided diminution in weight, without any other appreciable effect. In one case, the loss was from twelve to fifteen pounds in about two months; in another, the diminution in weight was thirty pounds in three months; and a third, there was a decided relief from the discomfort under which the patient had long labored, but the record is incomplete. The remedy is best administered in the form of alcoholic extract, of which there may be taken as much as three or four grammes daily. Great care should be used in the collection of the fucus, as it is easily confounded with other marine plants, which are entirely inert, so far as the peculiar efficacy of the vesiculosus is concerned.—*Boston Med. Jour.*

---

*Tetanus successfully treated by Chloride of Barium.*—The *Gazette des Hopitaux* reprints from the *Italian Medical Gazette* the details of a case of tetanus, in which the chloride of barium was successfully administered. It was prescribed in the proportion of sixteen grains to a pint of distilled water, the whole to be taken in the course of twenty-four hours. At the end of the eleventh day the symptoms had nearly ceased, and the dose was reduced to eight grains a day; on the sixteenth day the medicine was discontinued, and the patient left the hospital, well, on the eighteenth day. Several other cases of tetanus are referred to, in which this remedy has been employed with success in Italy, during the past two years.—*Boston Med. Jour.*

---

*Poisoning by Strychnia.*—Dr. Julian Harman reports, in the *Boston Journal*, May 1st, a case of poisoning by strychnia. Something like thirteen grs. of pure crystalized strychnia were taken in all. It was taken in two portions after dinner. Severe spasms ensued, which were quieted by Chloroform. An emetic was administered, and after its action the Chloroform was renewed. "At 7 o'clock, I gave six grains of Iodide of Potassium, with four drops of Tincture of Iodine; at 9, gave five grs. of Calomel, with one-third of a grain ot the Sulphate of Morphia, followed by Salts and Senna in the morning. During the night there was great muscular soreness, burning heat of the stomach and bowels, and vomiting of ropy fluid, which gradually abated, till in a few days the patient was well."

The Doctor observes that the mingling of the poison with the food prevented rapid absorption, and that a portion of it was probably ejected by vomiting. The Iod. Pot. had probably little effect in neutralizing the poison, "but the Camphor and Chloroform were probably far more effective than would have been the freest use of the best old Bourbon!"

It will be recollected that previous to the execution of Gordon, the slave captain, it was said that he had attempted his own life with Strychnia, which, whether true or false, was made the excuse for intoxicating him with Whiskey to the hanging point.

---

*Bromide of Potassium in Epilepsy.*—Several cases have been recently reported of successful treatment of Epilepsy by Bromide of Potassium. The usual dose is about three grains three times a day. It will probably be found to have about the average success of other empirical remedies for this variable and often intractable affection. It is suggested as particularly useful in cases connected with, or dependent upon, disorder of the sexual apparatus. The Bromide seems antaphrodisiac.

---

*Essays.*—Dr. John P. Spooner, of Dorchester, Mass., is publishing a series of papers in the *Boston Journal*, on "The Different Modes of Treating Disease." We trust that he will combine them and issue in a more permanent form. A good book on this subject at the present time is a desideratum.

---

*Podophyllum Peltatum.*—This vegetable threatens to displace a variety of hobbies just now. European writers have taken it up, and some of them have grown enthusiastic. The idea is advanced that it is about the only reliable chologogue in existence. It is asserted to be antisyphilitic beyond Mercury and Iodine. That it will promote or restrain discharges according as the indications are for one or the other, &c., &c.

We congratulate our hobby-riding friends on the cheapness and abundance of the new panacea. In this respect it is far ahead of Verat. Virid. and Chlorate of Potash. Having used it often within the last sixteen years, we are free to say, that it is the best indigenous substitute for Jalap of any substance with which we are acquainted. Its fresh root will prove actively emetic as well as cathartic—when dried it seems to lose emetic power. As an alterative it will rank with Sarsaparilla.

---

*Surgeon General of the U. S. A.*—The medical profession of the country are honored in the selection of Prof. Wm. A. Hammond, of the University of Maryland, as the new head of the Medical Department of the Army. He is well known as the author of numerous and valuable contributions to our periodical medical literature, and as occupying a front rank among experimental physiologists.

We believe he was formerly, for some ten or twelve years, an assistant surgeon in the regular army, resigning the position two or three years since. His executive qualities are said to be of a very high order, and his patriotism has been proved unimpeachable. In his present position he has an opportunity for exercise of high faculties of mind and practical knowledge, unequalled since the meridional days of Baron Lawcy.

---

*Chopart's Operation.*—Douglass Bly, M. D., reports to the N. Y. Medical Society: "The operation known as Chopart's, severs the flexors of the foot, and should *never* be performed under any circumstances whatever. The moment the flexors are severed, the extensors, having no antagonists, extend the foot on the leg, and cause the amputated surface to point almost directly downward. This deprives the patient of all power to use the remaining portion of the foot, and also renders him incapable of wearing a useful substitute. I am aware that, to obviate this difficulty, some surgeons have severed the tendo-achillis, but that has proved ineffectual, and it is only a partial relief at best. Therefore, amputation at this point renders the patient a hopeless cripple. The wound is slow to heal, *always tender*, often ulcerating, and the remaining portion of the foot is generally a curse to the patient as long as he lives, unless he submits to a secondary amputation."

---

*Treatment of Whitlow.*—All the asserted abortive methods of treatment are almost always useless. Those which can be relied on are the local antiphlogistics and cataplasms of mercurial ointment. They will at least moderate and limit the suppuration. The only really abortive treatment which can arrest the progress of the disease, is by early incisions, made where the pain is most severe, extended through the length of the inflamed part and deep in proportion to the gravity of the disease. When suppuration has commenced, incisions are indispensable. The bistoury is plunged down to the pus, to the tendinous sheaths or the periosteum, if they are implicated, and the tumor divided in its whole length. If the pain returns, it must be followed up with the bistoury, wherever it appears. After the incision, the hand should be kept for some time in warm water, or a poultice be applied ; the wound is afterward dressed with lint and cerate. Constitutional symptoms should be treated by diet and emollient drinks. Emetics and purgatives are indicated by symptoms of gastric or intestinal disturbance, and would also be useful as revulsives. Complications and sequelæ must be treated by appropriate measures.—*Dr. Laurel.*

---

*Tannin as Substitute for Peruvian Bark.—M. Leriche* in a recent memoir, awarded a prize by the Society of Medical Sciences of Brussels, revives the use of Tannin as an antiperiodic, but says it must be given in larger doses than formerly. Whatever the type of the periodic fever he recommends that the Tannin should be given in from twenty to thirty grain doses before the paroxysm. Two or three doses are usually sufficient to effect a cure. If the fever should not yield, fifteen grains only should be given in a mixture to be taken a table spoonful every hour. He has never seen the remedy fail in its effects. He has administered Tannin in 144 cases, ten of which were still under treatment at the time of publishing his memoir. 134 had been entirely cured, and two of these were men who had recently returned from Algiers with the African fever.

This treatment reminds of the gelatine cure of old time—but the question arises—when do the bowels get open ?

---

*Cubebs in Simple Urethritis of Women.—M. Trousseau* remarking upon the frequency of simple urethritis both in young girls and married women, " characterized by a frequen- desire to make water, with severe smarting during micturition, and vesical tenesmus lasting some minutes afterwards," commends freshly powdered cubebs as remarkably efficient. He advises from half a dram to a dram, twice a day at meals. "It should be continued several days, and as long as the symptoms last; when improvement begins the cubebs are given only once a day for a week, and in the following week, if improvement continues, the cubebs are given only once every second day."

Many cases of this kind, and even of gonorrhœal urethritis, where the cubebs fails to relieve, or disagrees with the patient, will be speedily ameliorated by moderate doses of *Abietis balsameæ*, which may be rendered not unpalatable by solution of Glycerine, or Mucilage and Sugar with Sp. Lavand. Comp. Where balsamic remedies are indicated, this particular one will be found very efficient, and less liable to produce gastric derangement, headache, &c., than those in more frequent use.

---

*Epidemic Influenza.*—In an epidemic influenza, characterized by profuse secretion of mucus and spasmodic coughing, sometimes simulating whooping cough, Dr. H. Bradford, of Nebraska, found the following mixture, especially beneficial: ℞ Syrup. Prun. Virgin. ℥ IV: Vin. Ipecac., Vin. Antim. aa ʒ ss; Tinct. Cannab. Indic ʒ j; Acid, Gallic, ʒ ss. M. A tea-spoonful every three or four hours.

---

*Causes af Tuberculosis.—Dr. Woody*, in a recent paper writes sensibly as follows:

"I would place at the head of the *causes* of consumption those influences in our lives that inevitably produce effeminacy: such as naturally result from the fashionable mode of living. The nervous exhaustion that follows in the train of indolence, the use of warm relaxing drinks such as coffee, tea, etc., the use of tobacco and other narcotics, that make heavy draughts on the brain, the sleeping on feather beds in close seven-by-nine rooms, and sexual excesses, constitute, I think, nine-tenths of the exciting causes of this disease."

Dr. W. thinks that in the young, of both sexes, masturbation is a predisposing and exciting cause that exceeds all others put together. Our own opinion, formed after many years careful observation, is that the number addicted to this disgusting vice is vastly over-estimated, and its alleged disastrous effects *hugely* exaggerated. The advertising columns of the newspapers afford ample evidence of the spoils which accrue to the nostrum venders, "Howard Associations," *et id genus omne*, from promulgation of such gross notions upon this subject. That occasional instances of fearful effect from this vice do occur is undeniable, but nothing in comparison to the swepeing statements so often made in the premises. It is safe to say that half of the cases of spermatorrhœa, &c., charged so often upon a long discontinued and repented vice, in fact depend upon dyspepsia, or upon the wrong habits of living to which Dr. W. has alluded in the above quotation.

We have in mind the case of a gentleman who came to us with a trifling *Herpes preputialis* which, with deep contrition, he confessed as the probable result of an illicit *coitus* five years previous, although it had only appeared two days before. Everybody knows such cases are the invariable dupes of the advertising knaves who "bleed" without letting of blood, and "cure without mercury." Add now, to cases of this sort, those who long since recollect to have rarely indulged in solitary vice, and let the terrible catalogue of resulting ills be instilled into their minds by such broad assevertions as that above, and the resulting moral, mental, and physical injury can scarcely be measured.

---

*Injections for Gonorrhœa.*—Dr. Bumstead, in his valuable treatise on venereal diseases, recommends (p. 57) that previous to injections for gonorrhœa, micturition should be performed, to clear the urethra of matter and to empty the bladder, so that the virus may not be carried before the injected fluid into the deeper parts of the passage, and that there may be no necessity of again evacuating the urine before the medicated fluid has had its full influence, and the temporarily augmented sensibility has abated. He suggests that it is better that quarter of an hour should elapse subsequent to passing the water, before use of the injection. In our opinion not more than five minutes should be allowed, for in ten or fifteen minutes, in very many cases at all events, the discharge will be found free on the urethral surface, and will tend first to neutralize the medicament, and, again, to be carried into the deeper parts.

This is another of the "little things," but may often determine the speedy cure or obstinate continuance of the disease.

---

*Bicarbonate of Potassæ in Croup.*—The *Druggist's Circular* states: Since Dr. Luscinsky, of Vienna, has recommended the bicarb. of soda, Dr. Kellogg has succeeded in establishing the claims of the bicarbonate of potassa, used by himself and others for a long time with decided success. After giving an emetic, he prescribes: ℞ Bicarb. Potassæ ʒ ss; Aq. Fœniculi ℥ v; Syrup. Senegæ ℥ j. M. A teaspoonful every half hour. In very severe cases he augments the dose to a grain and a half of the salt every quarter or half hour, and sometimes repeats the emetic." This treatment has at least the recommendation that it is a prophylactic against the injurious heroic methods sometimes adopted.

---

*Bebeerine in Menorrhagia.*—The *Reporter* recommends: "℞ Sulphate of Bebeerine gr. xij; Pulv. Cinnamon gr. xviij; Pulv. Ferri gr. vj. M. Divide in twelve parts. One to be taken every other day during the interval between the menstrual terms. During the flow, especially if it be profuse, the remedy should be given oftener and in larger doses."

We can readily believe the latter statement—a grain of Sulph. Bebeerine, with a grain and a half of Cinnamon and a grain of powdered Iron once in two days would hardly seem competent to control a particularly severe case of menorrhagia. The formula is commended to brethren with homœopathic tendencies as little liable to do harm, even though it does no good. Multiply the frequency of administration by six, at least, and it may prove beneficial in some cases.

*Invagination of the Intestine.*—In a late discussion in the Royal Med. and Chirurg. Soc., Dr. Stewart remarked that he had long since given up the use of purgatives in suspected cases of invagination of the intestines. He is now in the habit of using three or four grains of Extract of Belladonna in a large quantity of water by injection. In one case by mistake this injection was repeated but the poisonous effects were not developed. He had only seen two cases of delirium out of twelve thus treated. He had found the method usually successful.

Dr. S. observed that he resorted to the same plan in cases of poisoning by lead, in which colic and constipation were prominent symptoms.

This is vastly better than the old plan, but sometimes the Belladonna will prove unmanageable and it is variable in its strength. Laudanum or Morphine in large doses by injection we have seen frequently successful even in apparently desperate cases. Meanwhile let it be observed.

---

*Aneurism of the Extremities Treated by Flexion of the Limb.* This subject has recently been attracting considerable attention. Several successful cases are reported in the *London Lancet* and other journals. They were for the most part popliteal. The principle upon which the treatment is based is simple. Strong flexion of the limb considerably retards the blood in the main artery, and nearly arrests the pulse. The object in treating aneurism is not to altogether arrest the circulation in it, but to produce such a degree of retardation as will permit the formation of concentric fibrous laminæ on its interior. Absolute arrest of the blood is uncertain, and somewhat dangerous, in result. Retardation is safe and the effect permanent. Cures from flexion of the limb, even in popliteal aneurism, had been secured in six days, although, as a matter of precaution, semi-flexion is advised for several days subsequently. No instruments are needed, a bandage being the only requisite. Attendants are dispensed with, and, aside from the fatigue of the position, there is little to annoy or distress the patient. Pressure may sometimes be conjoined with advantage. The subject is called to the attention of the profession by Earnest Hart, Esq., Surgeon to the West London Hospital, whose first paper on the topic appeared in 1859. It is a method of rare interest both for its success and simplicity, and the sound physiological ideas upon which it is based.

---

*Ligation of the Funis.* That very sagacious obstetrician, Blundell, frequently deprecates the sarcasm: *Parva leves animos capiunt*, as applied to the little things necessary to be known and attended to by the practical accoucheur. Among these little things, our attention is directed to one in particular, by a passage on page 368 of Prof. Bedford's second edition of his valuable treatise on the Prin. and Prac. of Obstetrics. He proposes but one ligature, and says that he perceives no solid reason for two. He very properly observes, that the argument usually advanced in its favor—the liability of the mother to the hazards of flooding through the untied extremity of the cord—is founded on a false hypothesis, as he had previously pointed out when describing the anatomical arrangement of the placenta, and the fœtal circulation.

He remarks that:

"1. Two are unnecessary, because the small quantity of blood, which flows from the united extremity of the cord, consists merely of the disgorgement of the vessels on the fœtal surface of the afterbirth, and does not come directly from the system of the mother. 2. This very disgorgement, in my opinion, assists in the more prompt expulsion of the placenta."

We admit the fallacy, to a certain extent, of the reason for two ligatures which he adverts to, nevertheless we think that there are valid and important reasons for two ligatures.

1. Two ligatures are more cleanly. The untied extremity of the cord almost invariably distributes considerable blood, which, wherever it comes from, makes as disagreeable a "*muss*" of the surroundings as though it came from the uterine sinuses direct. This is almost entirely obviated by the use of two ligatures instead of one.

2. The retention of the blood in the placenta by the second ligature maintains its bulk to such an extent that the tendency to inertia of the uterus, the principal cause of delay in expulsion of the afterbirth, is very materially lessened. By this means uterine contraction is favored, speedy expulsion of the placenta, membranes and coagula is secured, and hæmorhage prevented, not solely from the vessels of the fœtal surface of the placenta, but from the otherwise inert and uncontracted uterus.

3. The distended placenta is the natural stimulus of the uterine contraction which thrusts it out from the cavity, and by securing its full distension by two ligatures of the funis, it will rarely ever be found necessary either to introduce the hand, or even finger, to excite, direct or reflex movements of the uterine muscle.

In the earlier years of the writer's practice, he employed but one ligature, and, as a result, often had to resort to the usual mechanical and medicinal measures for removing the "retained" placenta. But since adopting invariably the two ligatures, the instances where the placenta remains unexpelled, more than a few minutes, have been few and far between.

It may be remarked that the crushing of the vessels of the cord by the lower animals in biting off the funis, not only prevent bleeding from the young animal, but secures also the effect of the second ligature so far as the placenta is concerned. Its mass still remains so great that it is thrown off by the spontaneous action of the uterine muscles, without any necessity for either ergot or titillation of the internal surface of the cervix uteri by the finger.

We repeat this may be considered a little thing, but it is vastly more important, practically, than the treatment of *inversio uteri* or "hour-glass contraction." For these two the practitioner may spend a life of active practice without seeing

—whereas the other is to be attended to in every case of obstetrics.

*Camp Diarrhœa.*—The Army of the Union is suffering from the scourge of camps—diarrhœa, which, however, is troublesome by its disabling the men from duty, rather than from resulting mortality. There is no doubt that nineteen out of twenty cases might be prevented by attention to the plainest hygienic precepts.

It is presumed that the Southern Army are similarly afflicted, for an extract from a Memphis paper recently appeared among the daily telegraphic dispatches, setting forth that "Gen. Beauregard's effective force is essentially weakened by diseases peculiar to Mississippi bottoms." It is believed they are evacuating.

*Autumnal Diarrhœa, &c.*—Dr. Hines of Nottingham recommends, in the *London Lancet*, an old treatment: ℞ Ingus. Gent. comp. ℥ viij.; Tinct. Opi. ʒ iss.; Acid. Nitric gtt. XX. M. An ounce after every liquid stool or painful alvine evacuation. He uses at the same time sparing draughts of ice-cold mint tea.

### ANNUAL MEETING OF STATE MEDICAL SOCIETY.

Whereas the President, one Vice President, and the Chairman of most of the Standing Committees, together with a very large proportion of the members of the Illinois State Medical Society, are on active duty as Surgeons and assistant Surgeons with different divisions of the Army entirely beyond the limits of the State; and, whereas, many more are now, and will be for several weeks to come, engaged as volunteer Surgeons to aid in taking care of those wounded in the recent severe battles in Tennessee, therefore we, the undersigned, give notice that the Regular Annual Meeting of the State Society will be *postponed* until the first Tuesday in May, 1863.

Henry Gould.
Nath'l English.
Andrew McFarland.
Committee of Arrangements.

N. S. Davis, Permanent Sec'y.

*Clinical Lectures on the Diseases of Women and Children. By Gunning S. Bedford, A. M., M. D., Professor of Obstetrics, the Diseases of Women and Children, and Clinical Obstetrics, in the University of New York; author of "The Principles and Practice of Obstetrics." Medicus curat morbos, natura sanat. Hippocrates. Seventh Edition. Carefully revised. New York: William Wood,* 389 *Broadway.* 1862. *From the Author.*

This work is too well known and widely esteemed to render it necessary for us to do more than barely announce another edition.

The great variety of cases brought under review, give the work almost the characteristics of a systematic treatise, which it excels in interest by the life-like delineations that the clinical lecture gives opportunity to present.

The style which some reviewers have criticised, as rather too "free and easy" for a scientific work, has the advantage of interesting and sustaining attention, whilst the portraiture of diseases is so clear and distinct that he who runs may read.

Dr. Bedford never is obscure, his ideas are always transparent, and although here and there practitioners will differ as to his reasoning and methods, they will always know exactly what they differ from, so that there will be no occasion for *bush-fighting* in the matter.

We heartily commend the book to those of our readers who are not already possessed of it.

---

*Constitution, By-Laws and Code of Ethics of the Will County (Ill.) Medical Society. From Dr. G. S. Thomas, President of the Society.*

The objects of this association are well set forth in the preamble to the constitution:

"Inasmuch as an institution so conducted as to give frequent, united and emphatic expression to the views and aims of the medical profession in this county, must at all times have a beneficial influence, and supply more efficient means than have hitherto been available here for cultivating and advancing medical knowledge, for elevating the standard of medical education, for promoting the usefulness, honor and interest of the medical profession; for enlightening and directing public opinion in regard to the duties, responsibilities and requirements of medical men, for exciting and encouraging emulation and concert of action in the profession, and for facilitating and fostering friendly intercourse between those who are engaged in it, therefore be it *Resolved*, in behalf of the Medical Society of the County of Will, that for the organization and management of the same they adopt the following Regulations."

The Society is organized as auxiliary to the State Medical Society, and adopts the code of ethics adopted by that Society, at Springfield, June, 1850, any important violation of which is to be deemed sufficient reason of charge and expulsion. One very important By-Law of the Society we quote as suggestive:

"Sec. 8. Each member of this Society shall keep a record of all important cases that may occur in his practice, and such other facts as may fall under his observation, of especial interest to the profession, and report them to this Society and medical journals for publication."

We congratulate the Society on both its present condition and prospects.

---

*Hygiene of the Sewing Machine.* An advertisement of the widely known and justly celebrated sewing machine of Messrs. Wheeler & Wilson appears in the present number of the Journal, and we deem the occasion a fitting one in which to notice certain matters of hygienic importance strikingly connected therewith.

Since the fall of Adam, and the stitching of the primeval garment, a certain and gradually increasing amount of sewing has been required. Of one-half the race it has constituted the principal occupation, and unable to accomplish all demanded, a certain portion of the other half has been called upon to take part in the toil. Hence—tailors and man-milliners.

It is safe to say, that sewing has occupied more of human attention than any other branch of industry. Hood's "Song of the Shirt," wept over by so many, and which has stirred so many philanthropic souls to their very depths—touched but upon the very margin of the matter, the uttermost skirt of its garment—for more than half of the world was "stitch, stitch, stitching," with what of detriment to mind, body and soul it is worth while to think.

The tailor, who is but a temporarily hired Hessian in the great warfare against nakedness, is, by a melancholy adage, "the ninth part of a man"—what shall we say of woman, who, by the inevitable fiat of society, has been from time immemorial reduced to perpetual servitude to the needle?

The present writer thinks, for instance, that the so gravely mooted question of the psychological equality of the sexes can never be decided *pro* or *con* until the women receive their "freedom papers" from this needle-thraldom. There are some three or four millions of colored people on this continent who are obliged to have other people take the trouble to provide for their continuance in existence, and much concern does it give to very many people of humanitarian tendencies, and very much distraction to many other people not altogether humanitarians, but perhaps otherwise.

The needle has some hundreds of millions of slaves even now, although Wheeler & Wilson and some others have put into material shape an edict of emancipation which ought to have caused their crowning with laurels more worthily won than those of Clarkson, Wilberforce or John Brown.

The sewing machine is an organized protest against a slavery more intolerable than that of the Genius to Aladdin's lamp. It accomplishes more for the elevation of the feminine portion of the race, (and mediately of the other half) than did all the chivalry of the middle ages with its troops of gallant knights and squires.

But our space will not permit further allusion to its politico-economical, social or poetical, religious or psychological relations, we purposed to refer simply to its hygienic influences.

In an article read by A. K. Gardiner, M. D., Professor of Clinical Midwifery, and Diseases of Females, in the N. Y. Med. College, before the N. Y. Academy of Medicine. He asserts broadly, and proves by carefully collated statistical facts, that during an experience of ten years, the health of operators upon sewing machines of all varieties, was in no respect impaired by working upon them. In the factories, where many of the machines were used, not a single case could be found where injurious results could be traced to constant working upon the machines, although some of the girls "worked upon them for a year without losing a single day."

Contrast this with the results of constant, or even occasional use of the old fashioned hand needle, as observed in any neighborhood, even in the country. The dyspepsias, constipation, neuralgia, uterine affections, coughs, emaciation—phthisis!

We extract a couple of paragraphs from Prof. Gardiner's paper:

"The principal diseases said to be caused by the sewing machine are the so-called "female diseases" and spinal complaints. I have had some practice in these diseases, and may be allowed as a matter of personal experience to state that I have never seen a single patient who gained her living by working a sewing machine, who was affected by leucorrhœa, "falling of the womb," "ulcerations of the womb," or spinal difficulty—who ever had an abortion while using it, or who in any way could trace any injury from it. Neither have I had any patients in private practice with any diseases at all attributable to it. I have had many patients who have made up their family and children's clothing for the season, and their "baby linen" just before their lying in, with no injurious effects.

I am aware that the jar of the machine, and the "up and down" vibratory motion are stated to produce abortions, but this seems to be a most erroneous opinion, inasmuch as the "jar" of the machine, if there is any, falls not upon the feet or lower extremities, in which it is not felt in the slightest degree, but entirely upon the arms of the operator, resting upon the table; and from this undeniable reason, the alleged analogy between the hypothetical statement, that "the vitality of hen's eggs carried in cars, and subject to their vibratory and oscilating movement, is so destroyed that not one in a score will hatch," does not hold good, even if it can be proved that the human ovum in a healthy uterus is killed by this trembling movement, as is claimed by some. Upon this point, I have also a word to say in a proper place. Overwork, and by one unaccustomed or disused to the sewing machine, may, very probably, in some cases produce an abortion, and so will a long walk in the Central Park, a day's shopping, excessive laughing, even the eating of a bunch of grapes; yet shall these be denied the parturient woman? Shall we take the exception for the rule?"

Another objection which has been urged, is that the sewing machine injures the eyes of the operator. This has been fully negatived by the opinions based on large experience of distinguished oculists, among whom may be mentioned Dr. Geo. A. Bethune, of the Mass. Eye and Ear Infirmary, and Dr. Isaac Hays of Philadelphia, Drs. Wilkes, Clark, Ceccarini, and Stephenson of New York City, and Dr. Clark of Newark.

The "glass foot" which Messrs. W. & W. have added to their machine, does away entirely even with the theoretic objections upon this point.

The conclusion of Prof. Gardiner's paper is worthy of the careful consideration of every practitioner:

"A body in vigorous health is less liable to be seized with, or prostrated by, disease than a body in an atonic condition. If this is true of the whole frame, it is true of a portion of it, or of a single organ. The sewing machine overworks, that is wearies and fatigues, the learner, who exerts a muscular force, and for a too prolonged period, sufficient to drive half a dozen machines. The same instrument is but a healthy stimulant to the muscles of the lower extremities, of those accustomed to its use, developing and strengthening them. But the benefit and increased volume of the muscles actually employed is extended to the adjacent parts of the frame, and the muscles which belong to the pelvis, the back, and which support the abdominal walls, are called upon to aid in the work by steadying the frame, and firmly holding the parts to which the muscles of the lower extremity are attached. The developement of these muscles affects all the adjacent organs. The circulations are carried on more regularly, the absorbents are brought to work more energetically, and there is a tonicity very perceptible throughout the abdominal parietes, which is a result of the employment of the neighboring organs. In the female we have as a direct result, a "tone" in the generative apparatus before unknown, and a direct result of normal activity. The flaccidity of the vaginal walls is supplanted by contractility; the relaxed ligaments of the uterus become tense; the perineal muscles are developed; prolapsus uteri is impossible; leucorrhœas are absent, because dependent upon debility, malposition, and displacements; the secretions are normal, because the parts are in a normal condition. Now this is not theoretical, or at least is only the theory for the explanation of absolute facts which have come under the observation of myself, or of those who were well capable to judge; and who have communicated them to me. They may, perhaps, be called "coincidences," but the pustules upon the skin are also coincidences which, with others, make up what we denominate small-pox; and the coincidences which I shall proceed to relate may be found to be as marked and persistent as the variola cicatrices.

A case has been reported to me by a member of the Academy, of aggravated uterine disease, accompanied by prolapsus and leucorrhœa, which was of many years standing, and which had resisted all treatment, including pessaries, and other local applications, which was cured entirely and solely by the result of systematic and vigorous muscular exercise, united with healthy diet, and stimulating mountain air. Such cases are not unfrequent. Passive motion in a part produces a circulation of the stagnated blood, in its enlarged, congested vessels, and in their diseased condition is, perhaps, all the stimulus that they can bear. Active exercise or motion is only compatible with a comparatively healthy condition of these organs.

We will not seek to develope this view, but be content with its simple suggestion. But while the trundle-hoop, dumb-bells, and gymnastics generally, which have no result other than increased vigor of body, are recommended as prophylactics and invigorators, either partial or general, the exercise required in working a sewing machine should not be disregarded, especially as in addition to increased health, the pecuniary returns are worthy of consideration."

Combine now with this that open air exercise which the abbreviated hours of labor gives the sempstress opportunity to enjoy—let what work is done be done in salubrious, well ventilated rooms—let her cease to wear the the outrageous contrivances for beautifying (!) the form, which so often weigh down and oppress with their abnormal temperature the organs of the back and pelvis—give her good nutritious diet which, even if poor a few hours work with the "sewing piano" will enable her to procure, and in a brief period one of the largest classes of obstinate diseases with which the physician has to cope will disappear from daily observation, and scarcely be known, save in the books, as an altogether historical matter.

And the practitioner will in the end be a gainer, for this class of cases is largely made up of those who have exhausted his time and skill and patience with exceeding small returns of *honoraria.* The *opprobria medicinæ* will be lessened in number, and consequently the respectability of the profession enhanced.

We look upon the sewing machine as a grand auxiliary to the true physician, worth infinitely more to him than a whole pharmacopœia of new drugs which will lessen the number of the heart's beats per minute in the most marvellous degree, or which will "oxygenate the blood" in a manner most mysterious in extent and wonderful to behold.

---

*The War and Homœopathy.*—There is at least one good thing towards the bottom of the Pandora's box of evils which the great war has opened. For now the sick of the half million or so of men in the army are collected in masses where they can be observed by many at once, instead of being sparsely scattered hundreds and thousands of miles apart. The effects of treatment cannot be disguised. Abstract theories are brought to observable practical tests, and visionary speculations have to assume tangible form. The ignorance which, diluted and scattered over the wide superfices of private practice, could with difficulty be detected and satisfactorily exposed, in the compact throng of the army sick, is dragged from its obscurity and made glaringly obvious. The thin disguises of knavery and shallow pretension have been stripped off, and the chaff has been blown away from the wheat in the most satisfactory manner. In nothing is this more obvious than with regard to the attempts made by the homœopathists to foist their creatures upon the soldiery in the guise of medical attendants. Eight or ten months ago and they were rampant. They besieged the departments, protested in newspapers, assailed the President, and chose Mr. Senator Grimes, of Iowa, as a fitting conduit through which to pour out their noisome nonsense upon the Senate. They took the Commander-in-Chief captive, and kept the national army at a stand-still for four mortal weeks, whilst the Confederates threatened the Capitol, and they were titillating his palate with pellets of *Saccharum lactis* and teaspoonfuls of infinitesimal dilutions "chemically pure," with the identical teaspoon reposing on the top of the tumbler with its axis parallel to the meridian, like the wooden guns upon the Manassas fortifications, innocent and oblivious.

Here and there one of their disciples floated into the army disguised as a "regular"—and great was the jubilation of the Liliputians thereat. The newspapers puffed them, and even the God-fearing "Tribune," of this city, blew a nasal blast in token of rejoicing, much to the detriment of bystanders.

To-day, throughout the entire extent of the grand army, there is scarcely one so poor as to do them reverence. The departments at Washington are freed from their solicitations, the President from their impertinences, the Senate from their disturbing clamor, and even the Commander-in-Chief sends to Albany for Professor March to attend to his contusions, instead of telegraphing to New York for *Marcy*.

Homœopathy attempted the army and has found its Waterloo. It must henceforth conceal itself in the shades of private life—continue to dance attendance upon dyspeptic parsons, vaporous dowagers, and neuralgic spinsters. The atmosphere of the camps is too harsh for its delicate tissues, and the poor moth must quietly prey upon the curtains and carpets of a *parvenu* aristocracy, and not tempt destruction by approach to the torch of military glory. We commend its silence, and now suggest that it get itself decently buried before the army returns to dig for it an unhonored grave.

---

*Diseased Milk.*—The New York Legislature has recently passed an act to prevent the sale of impure, unwholesome or adulterated milk. The statute is a stringent one, and its penalties of such a character that its violation will be a matter of serious personal risk to the criminal. The "swill milk" business appears about to be done away with in New York. Much credit is due to the parties who have labored assiduously to abate this filthy and deadly nuisance. We hope to see other States following the example of New York, thus putting an end to the annual "Slaughter of the Innocents," which the nefarious traffic has brought about.

---

*Hygienic Measures in the Army.*—An eastern contemporary permits the use of its editorial columns by some anonymous writer to attack the editor of the Philadelphia Reporter, a job which the responsible conductors of the first mentioned paper have still too much self-respect to indulge in. Accordingly they put forward some other ☞ to do it. This ☞ man is evidently a "hot-livered grammarian," and "nothing if not critical." He probably travels at the expense of the Sanitary Commission, and writes under the potent influence of the prophylactics they so urgently recommended. The editor of the *Reporter* is amply able to take care of himself, even though this care may require the utter demolition of his assailant. All that we have to say about the matter is, that ☞ manifests a degree of ignorance of the so-called malarious diseases which, in a writer for a metropolitan monthly, is something more than deplorable. It is clear that he has not the slightest *practical* acquaintance with the class of diseases referred to, but has gathered all his notions at second hand from mere theorists. He declares that the plain and obvious hygienic measures, which *only* will prevent the incursions of "malarious" disease, are "utterly unattainable by an army in the field," and hence resort must inevitably be had to continuous dosing with Quinine as a prophylactic.

In the first place the record of many regiments in the field will show that these hygienic measures are very easily attainable, and the grand reason why "regulars" are far less liable to these diseases is, because their discipline more nearly secures their attainment.

The Quinine prophylaxis is, in the large proportion of cases, a mere dodge, or temporary expedient, to escape the necessary result of official laziness and incompetency, and private recklessness and insubordination.

The young gentleman quotes, with evident admiration, the remark of some one about the quinine theorizing "coming with tidings of joy and words of comfort to those who are subject to the influence of the poison." Is he so recent a neophyte in the medical ranks, as not to know that the very existence of such a "poison" is altogether hypothetical and speculative, and that, therefore, in the present time, treatment sought to be based upon such an idea is unworthy the age, and unworthy a scientific profession?

Diseases are not entities, attacking men as Indians do their victims; neither are the causes of diseases, save in rare instances, distinct and separable.

Disease is an orderly phenomenon, the resultant of all the forces acting upon the material of which the body is composed. Disease as the result of particular poisons is a phenomenon rarely seen—the search for the particular poisons which may beget disease, it is the glory of the profession at the present time, has been measurably merged in the investigation of larger, more pervading and general laws, the infringement of which brings disorder to the physical and psychical being.

Time was when disease was attributed to demoniac possession; then to witchcraft and enchantments; and then to specific poisons more or less occult, and then a large share of the medical world went off in vain quest of specifics, antidotes, counter poisons.

This "quinine prophylaxis" matter is but a shadow of the darkness of old time projected over upon the present, intensified by the same mode of support as the Catholicon, Morrison's Pills, or Brandreth's. "Instances confirmatory" can be gathered plentifully as blackberries. We remember when the whole North-west went mad over the same delusion and snare. Has the course of that empire gone around the globe to get to such distinction "down East?" For we speak what we do know when we say that, it has lost it sway where it originated—on the plains and along the watercourses of the North-west.

So far as appropriate hygienic measures are "attainable" in the field, so far will the army be freed from this class ot diseases. So far as they "are utterly unattainable by an army in the field"—so far will that army suffer from its ravages, and neither Morrison's nor Sappington's pills, neither Osgood's Indian Cholagogue nor Quinine will prevent.

Let the Homœopathists, the nostrum venders and quacks of other hues enter upon the wild goose chase for specifics and prophylactic drugs, but let no educated medical man prostitute his faculties to such a scarlet woman, or, rather Ixionic cloud.

---

*Researches and Observations on Pelvic Hœmatocele. By J. Byrne, M. D., M. R. C. S. E., Resident Fellow of the New York Academy of Medicine, &c. Monograph. From the Author.* 1862.

We had prepared a notice of this excellent monograph, but the unexpected amount of matter previously in type has crowded it over to the next number. The importance and interest of the subject is such that we are unwilling to condense our notice beyond the limits embraced in the article written. At present, therefore, we can merely thank the author and strongly commend his essay.

*An Address delivered before the Buffalo Medical Association, April 1st, 1862. By Dr. C. C. F. Gay, President of the Association, on retiring from the Chair. Published by vote of the Association.*

We have marked several paragraphs of this very able address which we shall take the liberty to transfer to subsequent pages of the JOURNAL.

The author will please accept our thanks for a copy. We congratulate the profession of our sister city on the excellent exposition of true medical science afforded in the address of their late presiding officer.

---

*Treatment of Diabetes.*—A communication by *M. Demneaux* to the Prussian Academy of Sciences, chronicles successful treatments of saccharine diabetes by equal parts of calcined alum and rhatany.

The alum, alone or with other astringents, has long been employed in the treatment of diabetes, but we think opium, or morphine, added largely, enhance its beneficial influence.

---

*Food in Febrile and Inflammatory Diseases.*—There comes a period in every inflammatory disease, when food of nutritious character becomes indispensable to successful management of the case. And this point, we are certain, is arrived at much earlier than frequently supposed. The appetite is by no means the test—neither emaciation nor extreme prostration. As soon as there are rational and physical evidences that the height of active exudation has been passed, thoroughly digestible and nutritious food should be given, even though anorexia or other symptoms of dyspepsia be present. In fact the greater portion of treatment, from that period, must be directed to the digestive derangement. Healthy blood is the best lotion, external or internal, which can be applied to a diseased part. In simple febrile affections the "feeding point" is earlier still. The expression, "living on *the fever*," is the rankest and most murderous nonsense ever uttered. Modern physiology and pathology have utterly exploded the old notions upon this subject.

